# Physical Activity Participation and Psychological Wellbeing in University Office Workers in China and Australia: An Online Survey

**DOI:** 10.3390/healthcare9121618

**Published:** 2021-11-23

**Authors:** Qian Sun, Meiling Qi, Wendy Moyle, Cindy Jones, Benjamin Weeks, Zihui Xie, Ping Li

**Affiliations:** 1 School of Physical Education, Shandong University, Jinan 250012, China; sunqian66@sdu.edu.cn; 2 School of Nursing and Rehabilitation, Shandong University, Jinan 250012, China; xzh_226@163.com; 3 Menzies Health Institute Queensland, Nathan Campus, Griffith University, Brisbane, QLD 4111, Australia; w.moyle@griffith.edu.au (W.M.); cjones@bond.edu.au (C.J.); b.weeks@griffith.edu.au (B.W.); 4 School of Nursing and Midwifery, Nathan Campus, Griffith University, Brisbane, QLD 4111, Australia; 5 Faculty of Health Science & Medicine, Bond University, Gold Coast, QLD 4226, Australia; 6 School of Health Sciences and Social Work, Gold Coast Campus, Griffith University, Gold Coast, QLD 4222, Australia

**Keywords:** health status, physical activity, university workers

## Abstract

*Background and purpose:* Physical inactivity and prolonged sitting have associations with adverse health. University office workers are at a high risk of psychological and pain issues. This study aimed to explore the differences in sitting time, levels of physical activity participation, stress, depression, anxiety, and pain among university office workers in China and Australia. *Methods*: Online surveys were distributed to university office workers over 55 years at two universities in China and Australia, respectively. *Results*: A total of 185 participants completed the online survey (119 in China and 66 in Australia). Significant differences were found in sitting time during workdays between the two countries (*p* < 0.05) with a longer sitting time in the Australian respondents (7.5 h/day) than those in China (4.6 h/day). Additionally, there were also significant differences in terms of levels of depression and pain symptoms within the two countries (*p* < 0.05). The Australian respondents reported high levels of depression and pain (M = 7.38, SD = 5.86 and M = 3.65, SD = 2.21, respectively) than those in China (M = 5.71, SD = 4.87 and M = 1.89, SD = 1.89, respectively). The gender, education level, and sitting time of participants were found to be associated with pain scores (*p* < 0.05). A significant association between marital status and pain scores was found among the Australian respondents (*p* < 0.05). *Conclusions*: Future studies with a larger population are needed to validate the results and to further explore the association between physical activity participation and psychological wellbeing among university office workers.

## 1. Introduction

Physical inactivity is a modifiable risk factor for a range of chronic diseases among middle-aged and older adults including obesity and cardiovascular disease [1]. There is also a strong association with prolonged sitting time at work, particularly among university office workers [2]. Work stress, in association with aggravated competition and job insecurity, is considered to be a growing threat to the health of aging employees [3]. Chronic pain is also associated with reduced mobility, increased depression, and anxiety. Both depression and anxiety symptoms affect the performance of university office workers, resulting in poor concentration and work ability [4]. Importantly, neck pain has been reported to be a major health problem among computer-based workers [5]. To reduce the levels of stress, depression, anxiety, and pain, there has been an emphasis on the importance of increasing the participation of university office workers in physical activity whilst also reducing sitting time to improve health outcomes [6]. For example, a 12-week worksite aerobic physical activity intervention improved physical fitness and psychological wellbeing among the university workers employed at Kent State University, USA [7].

Employees at the University of Minnesota Duluth, USA, spent 75% of their workday seated with only 12% of them engaging in any walking activity [8]. Nearly half of the employees of one UK University reported physical activity below the recommended guidelines where females were particularly less active than males [6]. Both librarians and IT professionals in a Nigerian University reported work-related stress because of long periods of computer screentime, poor job incentives, and the development of constant neck pain [9]. In addition, 21.7% of academic and non-academic staff of University Putra Malaysia were exposed to a range of job stressors; for example, a high job demand and a lack of social support [10]. As for symptoms of anxiety and depression, a higher percentage of university staff showed clinical scores of anxiety and depression compared with the general population [11]. Almost half of the university office workers in Australia reported headache and neck pain in one of our previous studies [12]. Thus, of the international research to date on the physical activity and psychological health of university employees, studies have focused on sedentary sitting time, psychological wellbeing (e.g., stress, depression, and anxiety), and pain issues. However, minimal attention has been paid to a comparison of the above outcomes among university office workers between different countries [13].

Older adults contribute greatly to society with more than half of the people aged 55–64 years still engaged in the workplace in Australia [14]. Similarly, the retirement age in China has increased from 55 to 65 years and older [15]. An increase in the older labor workforce participation rate means that more attention should be paid to the health of older workers to ensure their physical and psychological health in the workplace of the two countries. However, different guidelines and services in mental condition prevention and physical activity participation may be likely between China and Australia [16]. Cultural factors such as perceptions of mental illness and socio-contextual factors (e.g., mental health care systems) also contribute to the differences in recognition of depression and other mental health conditions in China and Australia [17]. Different opportunities also exist for university office workers to effectively engage in physical activity including different exercise resources, fitness services, and costs [18]. For example, there is a higher percentage of university office workers engaged in casual employment in Australia than in China, which might also impact on their potential to partake in physical activity because of their irregular working hours [19]. Thus, university office workers in the two countries might have different levels of physical activity and psychological wellbeing due to different policies, health perceptions, and health care systems. To the best of our knowledge, no existing studies have compared physical activity participation, sedentary sitting, and psychological outcomes among the university office workers in China and Australia.

Given the authors are interested in the aging workforce, the decision was made to include potential participants aged 55 years and older. Due to the experience of prolonged sitting during working hours as well as work-related physical impairments, psychological issues, and chronic pain among older office workers, there is a need to pay more attention to this population. Hence, older office workers in this study are defined as academic and administrative staff aged 55 years and older who are employed by universities [12]. In order to inform the development of an appropriate workplace intervention that will positively affect the health of university office workers 55 years and older in China and Australia, the aim of this study was, therefore, to use an online survey to investigate the levels of physical activity participation, stress, depression, anxiety, and pain as well as the sitting time of university office workers from China and Australia in order to explore: (1) the differences in physical activity, sedentary time, and psychological wellbeing of university office workers over 55 years between the two countries; (2) the associations between the demographic characteristics and psychological wellbeing in the two countries; and (3) the associations between physical activity participation and sitting time and psychological wellbeing in the two countries. This study hypothesized that there would be significant differences in the physical activity participation and sitting time and the levels of stress, depression, anxiety, and pain as well as their associations between the two countries.

## 2. Materials and Methods

### 2.1. Participants and Ethical Considerations

This study recruited university office workers aged 55 years and older working at two universities in South East Queensland, Australia and Shandong province, China, respectively. Potential participants who were administrative or academic staff with no cognitive impairment and normal communication abilities were included in this study. The target sample size of the participants was not calculated and this study aimed to seek as many participants as possible to complete the survey. A total of 185 potential participants completed the survey including 119 respondents in China and 66 participants in Australia.

The participants were invited to complete an online survey that was administered via a free online Chinese survey platform (https://www.wjx.cn/, accessed on 10 February 2021) and Lime Survey software [20] in Australia. Lime Survey and the Chinese survey website are two free and open-source survey software tools that have been used by previous researchers and are valid for data collection among Chinese and non-Chinese populations over 55 years [20]. Potential participants in China were recruited through the WeChat mobile instant text communication service, which is the most utilized social media platform by the Chinese population. Emails seeking potential participants were sent to the university staff in Australia from the University Deputy Vice Chancellor (Administration) as part of the regular email correspondence to staff about research participation. Ethics approvals of the study were gained from the University Human Research Ethics Committee (2020-12-037 China, and 2016/448 Australia). The completion and submission of the online survey implied consent to participate in this study, which was declared to respondents at the welcome page of the online survey.

### 2.2. Data Collection and Measures

Data collection in Australia took place between July 2016 to August 2016. In China, data were collected from February 2021 to March 2021. The online survey included the demographic details (e.g., age, gender, education levels, and marital status) of the participants. Self-reported physical activity participation was assessed by the International Physical Activity Questionnaire Short Form (IPAQ-SF) [21]. The IPAQ-SF was used to collect the physical activity levels and sitting time of the participants during the previous seven days. In addition, the levels of perceived stress, depression, anxiety, and pain were assessed by the 10-item Perceived Stress Scale (PSS-10) [22], the 10-term Center for Epidemiological Studies Depression Scale (CES-D10) [23], the Geriatric Anxiety Inventory (GAI) [24], and the Visual Analogue Scale (VAS) [25]. Details of the measurements used among the Australian respondents are described in our previously published paper where they were reported to be reliable and valid measures for adults over 55 years to assess physical activity participation, sedentary sitting time, and psychological wellbeing [26]. Chinese versions of the above measurements were used with the Chinese participants. Studies have also shown that the Chinese versions of the IPAQ-SF, PSS-10, CES-D10, GAI, and VAS have a high reliability for use with the Chinese population aged over 55 years [21,27,28,29]. All questions were set as mandatory to encourage participants to answer all questions included in the surveys.

### 2.3. Statistical Analysis

The data analysis was conducted using the Statistical Package for Social Sciences (SPSS) version 26.0. The descriptive statistics were calculated using frequencies (i.e., percentage) for the categorical variables (i.e., age, gender, education level, and marital status) and mean and standard deviations for the continuous variables (i.e., physical activity, sitting time, stress, depression, anxiety, and pain). The chi-squared test was used to analyze the differences in the categorical variables between the two samples. The differences in the physical activity participation levels, sitting time, perceived stress, depression, anxiety, and pain between Australia and China were conducted using the independent *t*-test. A linear regression was conducted to calculate the bivariate associations between the demographic characteristics, levels of physical activity, sitting time, perceived stress, depression, anxiety, and pain for all participants. The significance level was set at 0.05.

A receiver operating characteristics (ROC) analysis was conducted to evaluate the accuracy of the significant differences in sitting time between the two countries. The area under the ROC curve (AUC), which jointly considers sensitivity and specificity, was calculated to assess the overall accuracy of this outcome. A ROC curve plots the false positive rate (1: specificity) on the x-axis and the true positive rate (sensitivity) on the y-axis. ROC-AUC values of ≥0.90, 0.80–0.90, 0.70–0.80, and <0.70 are considered to be excellent, good, fair, and poor, respectively [30].

## 3. Results

The characteristics of the participants are presented in Table 1. In both countries, most respondents (more than 86.4%) were aged between 55 and 64 years. Most of the recruited participants were married (89.9% in China and 60.6% in Australia). However, 33.3% of the participants in Australia were widowed, divorced, or separated compared with 1.7% of the participants in China (*p* < 0.05). A higher percentage of Australian respondents had less than a bachelor’s degree compared with the Chinese (*p* < 0.05) with 28.8% and 19.3% of the participants having less than a bachelor’s degree in Australia and China, respectively. As for the gender of the participants, most (86.4%) were female in Australia whereas 51.3% were female and 48.7% were male in China (*p* < 0.05).

**Figure 1:**
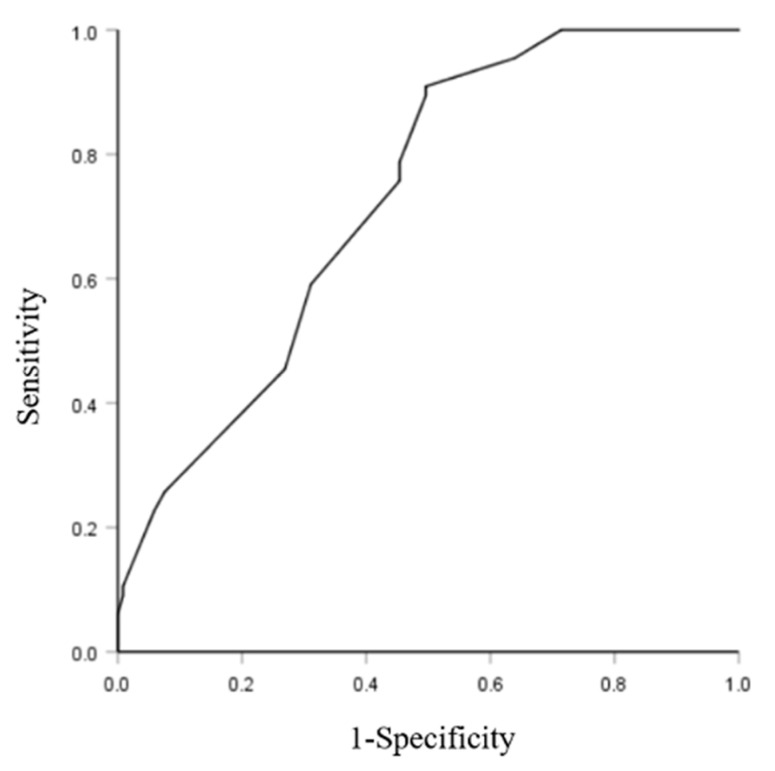
ROC curve for the sitting time.

Table 2 shows the physical activity participation, stress, depression, anxiety, and pain of the participants as well as the sitting time in both countries. Based on similar standard working hours of university office workers in China and Australia (i.e., 8 h and 7.5 h, respectively), significant differences were found in the sitting time during workdays in the previous seven days between the two countries (*p* < 0.05) with an average sitting time of 7.50 h (SD = 2.75) and 4.64 h (SD = 3.22) per day in Australia and China, respectively. A ROC value of 0.73 (i.e., fair) was found for the sitting time between the two countries, as shown in Figure 1. There were also significant differences in terms of the levels of depression and pain symptoms within the two countries (*p* < 0.05). The depression levels of the participants were significantly higher in Australia (M = 7.38, SD = 5.86) than in China (M = 5.71, SD = 4.87). Similarly, the participants reported higher levels of pain in Australia (M = 3.65, SD = 2.21) than in China (M = 1.89, SD = 1.89). No significant differences were found between the two countries in terms of physical activity participation, levels of stress, or anxiety.

For all the included 185 participants, the male gender and bachelor education level were significantly associated with lower pain scores (*p* < 0.05) (see Table 3). The sitting time of the participants was also found to have a positive association with the levels of pain (*p* < 0.05). No associations were found between the demographics and the levels of stress, depression, anxiety, and pain in China (see Table 4). However, married Australian university office workers experienced less pain than others (*p* < 0.05). Similarly, Australian respondents who were widowed, divorced, or separated also reported less pain (*p* < 0.05).

## 4. Discussion

This is the first study known of to compare physical activity, sedentary sitting, and psychological wellbeing of university office workers in China and Australia. No differences were found in the physical activity participation of the participants in the previous seven days between the two countries whereas participants in Australia had significantly longer sitting on workdays during the previous seven days than those in China. The study result also confirmed previous research [6,31] that the majority of university office workers have sedentary behavior on workdays. For example, a cohort study with 5246 Australian middle-aged participants in four survey waves also indicated that most of the participants spent more than 5 h sitting watching TV and at work [31]. The differences in sitting time between the two countries might suggest that researchers should pay more attention to the sedentary behavior of Australian university office workers and find more targeted strategies to reduce their sedentary sitting time. Importantly, no relationship was found between the physical activity participation and the levels of stress, depression, and anxiety as well as pain, which was not consistent with previous studies [6,7]. This was, however, consistent with our previously published article regarding physical activity and psychological wellbeing in Australian university office workers [12] where the small sample size, different measurement of psychological variables, and different characteristics of the study population were concluded to be the potential reasons for the differences in the outcomes. The sample of 185 participants in this study might make it difficult to find significant relationships from the data.

Importantly, the current study highlighted the negative association between sitting time and the levels of pain symptoms in university office workers. A longer duration of sitting time was found to be a risk factor for low back pain and has often been associated with discomfort and/or pain in the neck/shoulder regions [26,32,33]. Furthermore, a longer sitting time with less physical activity further increases the symptoms of pain. Thus, previous studies have suggested applying effective strategies (e.g., exercise interventions) that aim to reduce sedentary behavior and decrease pain symptoms among the workplace population [34,35]. However, due to the small sample size in the current study, the explanation of this association might need further confirmation in prospective studies using appropriate methods to determine a suitable sample size.

This study also implied that university office workers who had a higher degree than a bachelor’s degree experienced low levels of pain. Sultana [36] suggested that those who had higher levels of education had a greater awareness of pain symptoms and also understood the importance of seeking early interventions to manage pain. Thus, the diverging education levels between the two countries in the current study might explain the differences in the pain symptoms of university workers. Additionally, the current study found that Australian participants who were married/divorced/widowed reported low pain scores. This finding was consistent with a previous study [37], which reported that women who were married were significantly more likely to have lower scores of pain. The significant association between marital status and the levels of pain in the current study may suggest that companionship may improve pain perception, especially in Australians. Partners were the first preferred source of support because of their relational proximity [38]. A lack of support had a negative effect on the ability of people to cope with pain. For this reason, the support of a companion was thought to be useful in lessening the experience of pain [39].

There was a significant difference in the levels of depression among the participants in the two countries with lower levels of depression in China. Previous studies indicated that lower educational levels of older workers had an association with a greater lack of job control and depression [12,40]. Different education levels might be a potential reason for the different levels of depression between the two countries. However, no significant associations between the education level and pain could be detected in the current study, which might also be due to the small sample size. Other possible explanations for the differences may relate to different cultural factors and socio-economic statuses (e.g., the policy, health service, health perception, and health care system) as indicated in the introduction, which were also found to be strongly associated with depression and appeared to operate across the life course [17,41]. Future studies should investigate whether the education level, cultural, and socio-economic differences are important elements influencing the depression levels of university office workers in China and Australia.

The strength of this study was the comparison of both physical activity and wellbeing of university office workers in China and Australia. However, the study results, especially the association between physical activity and wellbeing, were different from published studies in university populations, potentially because of the small sample size, which may be worthy of future study. This study aimed to seek as many participants as possible to complete the survey. The relatively small sample size was a limitation of this study. Different sample sizes between the two countries may also limit the representativeness of the comparative results. Anthropometric data such as height, body weight, and body mass index (BMI) might have relationships with sedentary sitting, participation in physical activity, and psychological wellbeing. However, the missing collection of anthropometric data may be another limitation of this study. Importantly, the different periods of data collection in the two countries may also influence the study results as the public health emphasis on sitting and physical activity might have changed within a 5-year period in the two countries.

## 5. Conclusions

Australian university office workers reported a longer sitting time and higher levels of depression and pain issues. Sitting time can have a negative association with levels of pain. The findings may be useful in helping researchers and university administrators develop intervention programs to reduce sitting time and improve the mental health of university office workers. The results from the current study also suggest that the demographics of the participants (i.e., gender and educational level) might contribute to the different levels of pain between the participants in both countries. Importantly, the marital status was also associated with the levels of pain, especially in Australian university office workers. A larger study is needed to validate the results and further explore the associations between physical activity participation and the levels of stress, depression, anxiety, and pain in university office workers.

## Figures and Tables

**Table 1:** Demographic characteristics of the participants.

Demographic Variables	China (*n* = 119) *n* (%)	Australia (*n* = 66) *n* (%)	*χ^2^*	*p*
Age (years) 55–64 65 and above	104 (87.4%) 15 (12.6%)	57 (86.4%) 9 (13.6%)	0.00	1.00
Gender Female Male	61 (51.3%) 58 (48.7%)	57 (86.4%) 9 (13.6%)	21.15	0.00 *
Educational level Less than bachelor’s degree Bachelor’s degree Postgraduate degree	23 (19.3%) 43 (36.1%) 53 (44.5%)	19 (28.8%) 9 (13.6%) 38 (57.6%)	10.79	0.00 *
Marital status Single Married Other	10 (8.4%) 107 (89.9%) 2 (1.7%)	4 (6.1%) 40 (60.6%) 22 (33.3%)	37.69	0.00 *

* indicates significant differences between China and Australia (*p* < 0.05).

**Table 2:** Comparison of physical activity and other health variables between China and Australia.

Variable	China M (SD)	Australia M (SD)	*t*	*p*
Physical activity (MET-min/week)	1704 (2133)	1849 (1957)	0.46	0.65
Sitting time (h/day)	4.61 (3.22)	7.50 (2.75)	6.46	0.00 *
Stress	13.76 (5.44)	12.62 (8.00)	−1.04	0.30
Depression	5.71 (4.87)	7.38 (5.86)	2.08	0.04 *
Anxiety	3.80 (5.00)	3.45 (5.17)	−0.41	0.68
Pain	1.89 (1.89)	3.65 (2.21)	5.70	0.00 *

* indicates significant differences between China and Australia (*p* < 0.05); M: mean; SD: standard deviation; MET: metabolic equivalent.

**Table 3:** Associations between the demographics, physical activity participation, and sitting, and the levels of stress, depression, anxiety, and pain (*n* = 185).

Variables	Total (China and Australia)							
	Stress		Depression		Anxiety		Pain	
	*B*	*P*	*B*	*P*	*B*	*P*	*B*	*P*
Age								
55–64 (Constant)								
65–74	−0.06	0.41	−0.09	0.21	−0.06	0.39	−0.11	0.15
Gender								
Female (Constant)								
Male	0.04	0.59	0.03	0.67	−0.01	0.86	−0.24	0.00 *
Education level								
Less than bachelor’s degree (Constant)								
Bachelor’s degree	−0.09	0.34	−0.18	0.06	0.00	0.97	−0.24	0.01 *
Postgraduate degree	−0.10	0.27	−0.09	0.33	−0.07	0.44	−0.09	0.31
Marital status								
Single (Constant)								
Married	−0.02	0.88	−0.02	0.85	0.02	0.90	−0.09	0.44
Other	−0.03	0.80	0.11	0.31	0.01	0.93	0.04	0.71
Physical activity	−0.11	0.14	−0.05	0.48	−0.09	0.25	0.01	0.87
Sitting	0.05	0.51	0.09	0.21	0.10	0.20	0.16	0.03 *

* indicates significant differences (*p* < 0.05); *B*: standardized regression coefficient (Beta); *P*: *p*-value.

**Table 4:** Associations between the demographic variables, physical activity, and sitting, and the levels of stress, depression, anxiety, and pain in the Chinese and Australian participants.

Variables	China								Australia							
	Stress		Depression		Anxiety		Pain		Stress		Depression		Anxiety		Pain	
	*B*	*P*	*B*	*P*	*B*	*P*	*B*	*P*	*B*	*P*	*B*	*P*	*B*	*P*	*B*	*P*
Age																
55–64 (Constant)																
65–74	−0.16	0.0	−0.16	0.08	−0.11	0.22	−0.11	0.22	0.06	0.64	−0.00	0.98	0.03	0.79	−0.14	0.27
Gender																
Female (Constant)																
Male	0.02	0.80	0.15	0.10	0.01	0.88	−0.11	0.22	−0.01	0.94	−0.03	0.79	−0.14	0.27	−0.14	0.27
Education level																
Less than bachelor’s degree (Constant)																
Bachelor’s degree	−0.02	0.86	−0.0	0.70	0.05	0.70	−0.17	0.18	−0.21	0.14	−0.21	0.14	−0.15	0.30	−0.12	0.39
Postgraduate degree	−0.03	0.82	0.05	0.67	−0.08	0.52	−0.08	0.53	−0.17	0.23	−0.23	0.10	−0.06	0.68	−0.10	0.51
Marital status																
Single (Constant)																
Married	0.04	0.66	0.04	0.71	0.10	0.30	0.05	0.60	−0.18	0.48	−0.20	0.44	−0.40	0.13	−0.53	0.04 *
Other	−0.18	0.07	−0.05	0.66	−0.03	0.80	−0.04	0.68	−0.06	0.82	−0.04	0.88	−0.28	0.27	−0.58	0.03 *
Physical activity	−0.13	0.15	−0.00	0.99	−0.07	0.47	−0.01	0.90	−0.08	0.54	−0.15	0.22	−0.12	0.33	0.02	0.89
Sitting	0.03	0.72	−0.02	0.79	0.12	0.21	−0.01	0.88	0.19	0.14	0.14	0.27	0.13	0.29	0.02	0.88

* indicates significant differences (*p* < 0.05); *B* = standardized regression coefficient (Beta); *P*: *p*-value.

## Data Availability

The data presented in this study are available on request from the corresponding author.

## References

[1] Ewing R., Meakins G., Hamidi S., Nelson A.C. (2014). Relationship between urban sprawl and physical activity, obesity, and morbidity—Update and refinement. Health. Place.

[2] Thorp A.A., Healy G.N., Winkler E., Clark B.K., Gardiner P.A., Owen N., Dunstan D.W. (2012). Prolonged sedentary time and physical activity in workplace and non-work contexts: A cross-sectional study of office, customer service and call centre employees. Int. J. Behav. Nutr. Phys. Act..

[3] Hyun J., Sliwinski M.J., Almeida D.M., Smyth J.M., Scott S.B. (2017). The moderating effects of aging and cognitive abilities on the association between work stress and negative affect. Aging Ment. Health.

[4] Stynen D., Jansen N.W., Kant I.J. (2015). The impact of depression and diabetes mellitus on older workers’ functioning. J. Psychosom. Res..

[5] Kearney G.D., Allen D.L., Balanay J.A.G., Barry P. (2016). A descriptive study of body pain and work-related musculoskeletal disorders among latino farmworkers working on sweet potato farms in eastern North Carolina. J. Agromedicine.

[6] Cooper K., Barton G.C. (2016). An exploration of physical activity and wellbeing in university employees. Perspect. Public Health.

[7] Corbett D.B., Fennell C., Peroutky K., Kingsley J.D., Glickman E.L. (2018). The effects of a 12-week worksite physical activity intervention on anthropometric indices, blood pressure indices, and plasma biomarkers of cardiovascular disease risk among university employees. BMC. Res. Notes.

[8] Fountaine C.J., Piacentini M., Liguori G.A. (2014). Occupational sitting and physical activity among university employees. Int. J. Exerc. Sci..

[9] Ajala E.B. (2011). Work-related Stress among Librarians and Information Professionals in a Nigerian University. Libr. Philos. Pract..

[10] Mukosolu O., Ibrahim F., Rampal L.G., Ibrahim N. (2015). Prevalence of stress and its associated factors among university staff. Malays. J. Med. Health. Sci..

[11] Mark G., Smith A.P. (2012). Effects of occupational stress, job characteristics, coping, and attributional style on the mental health and job satisfaction of university employees. Anxiety Stress Coping.

[12] Qi M., Moyle W., Jones C., Weeks B. (2019). Physical Activity and Psychological Well-Being in Older University Office Workers: Survey Findings. Workplace Health Saf..

[13] Akpunne B.C., Uzonwanne F. (2017). Psychopathological symptoms among medical and non-medical leadership and supervisory employees of two University Teaching Hospitals. Int. J. Innov. Res. Dev..

[14] Australian Institute of Health and Welfare (2015). Australia’s Welfare 2015.

[15] Peng X., Mai Y. (2013). Population Ageing, Retirement Age Extension and Economic Growth in China a Dynamic General Equilibrium Analysis.

[16] Zhou S., Davison K., Qin F., Lin K.F., Zhao J.X. (2019). The roles of exercise professionals in the health care system: A comparison between Australia and China. J. Exerc. Sci. Fit..

[17] Wong D., Cheng C.W., Zhuang X.Y., Ng T.K., Pan S.M., He X., Poon A. (2017). Comparing the mental health literacy of Chinese people in Australia, China, Hong Kong and Taiwan: Implications for mental health promotion. Psychiatry Res..

[18] Bauman A., Bull F., Chey T., Craig C.L., Ainsworth B.E., Sallis J.F., Bowles H.R., Hagstromer M., Sjostrom M., Pratt M. (2009). The International Prevalence Study on Physical Activity: Results from 20 countries. Int. J. Behav. Nutr. Phys. Act..

[19] Hahn M.H., Mcvicar D., Wooden M. (2020). Is casual employment in Australia bad for workers’ health?. Occup. Environ. Med..

[20] Schmitz C. Limesurvey: An Open Source Survey Tool. http://www.limesurvey.org.

[21] Craig C.L., Marshall A.L., Sjostrom M., Bauman A.E., Booth M.L., Ainsworth B.E., Pratt M., Ekelund U.L.F., Yngve A., Sallis J.F. (2003). International Physical Activity Questionnaire: 12-Country Reliability and Validity. Med. Sci. Sports Exerc..

[22] Roberti J.W. (2006). Further psychometric support for the 10-item version of the perceived stress scale. J. Coll. Couns..

[23] Irwin M., Artin K.H., Oxman M.N. (1999). Screening for depression in the older adult: Criterion validity of the 10-item Center for Epidemiological Studies Depression Scale (CES-D). Arch. Intern. Med..

[24] Pachana N.A., Byrne G.J., Siddle H., Koloski N., Harley E., Arnold E. (2007). Development and validation of the Geriatric Anxiety Inventory. Int. Psychogeriatr. IPA.

[25] Phan N.Q., Blome C., Fritz F., Gerss J., Reich A., Ebata T., Augustin M., Szepietowski J.C., Ständer S. (2012). Assessment of pruritus intensity: Prospective study on validity and reliability of the visual analogue scale, numerical rating scale and verbal rating scale in 471 patients with chronic pruritus. Acta Derm. -Venereol..

[26] Machado L., Viana J.U., Silva S., Couto F., Mendes L.P., Ferreira P.H., Ferreira M.L., Dias J., Dias R.C. (2018). Correlates of a recent history of disabling low back pain in community-dwelling older persons. Clin. J. Pain.

[27] Leung D.Y., Lam T.H., Chan S.S. (2010). Three versions of Perceived Stress Scale: Validation in a sample of Chinese cardiac patients who smoke. BMC. Public Health.

[28] Huang Q., Wang X., Gong C. (2015). Reliability and Validity of 10-item CES-D among Middle Aged and Older Adults in China. China J. Health Psychol..

[29] Dow B., Lin X., Pachana N.A., Bryant C., Logiudice D., Anita M., Haralambous B. (2018). Reliability, concurrent validity, and cultural adaptation of the Geriatric Depression Scale and the Geriatric Anxiety Inventory for detecting depression and anxiety symptoms among older Chinese immigrants: An Australian study. Int. Psychogeriatr. IPA.

[30] Metz C.E. (1978). Basic principles of ROC analysis. Semin. Nucl. Med..

[31] Mielke G.I., Burton N.W., Gavin T., Brown W.J. (2018). Temporal trends in sitting time by domain in a cohort of mid-age Australian men and women. Maturitas.

[32] Smp A., Hjk A., Hj A., Hk B., Chang B.S., Lee C.K., Jin S. (2018). Longer sitting time and low physical activity are closely associated with chronic low back pain in population over 50 years of age: A cross-sectional study using the sixth Korea National Health and Nutrition Examination Survey. Spine J..

[33] Hallman D.M., Gupta N., Mathiassen S.E., Holtermann A. (2015). Association between objectively measured sitting time and neck–shoulder pain among blue-collar workers. Int. Arch. Occup. Environ. Health.

[34] Barone G.B., Hergenroeder A.L., Perdomo S.J., Kowalsky R.J., Delitto A., Jakicic J.M. (2018). Reducing sedentary behaviour to decrease chronic low back pain: The stand back randomised trial. Occup. Environ. Med..

[35] Pronk N.P., Katz A.S., Lowry M., Payfer J.R. (2012). Reducing occupational sitting time and improving worker health: The Take-a-Stand Project, 2011. Prev. Chronic Dis..

[36] Sultana R. (2015). Association between education and knowledge of maternal health among urban women in Dhaka city, Bangladesh. Eur. Acad. Res..

[37] Bjonnes A.K., Parry M., Lie I., Falk R., Leegaard M., RustoEn T. (2018). The association between hope, marital status, depression and persistent pain in men and women following cardiac surgery. BMC Women’s Health.

[38] Ariyo A.M., Mgbeokwii G.N. (2019). Perception of companionship in relation to marital satisfaction: A study of married men and women. IFE. Psychol..

[39] Ntombana R., Sindiwe J., Ntombodidi T. (2014). Opinions of labouring women about companionship in labour wards. Afr. J. Mid. Women’s Health.

[40] Haralambous B., Dow B., Goh A., Pachana N.A., Bryant C., LoGiudice D., Lin X. (2016). ‘Depression is not an illness. It’s up to you to make yourself happy’: Perceptions of Chinese health professionals and community workers about older Chinese immigrants’ experiences of depression and anxiety. Australas. J. Ageing.

[41] Dorner T.E., Muckenhuber J., Stronegger W.J., Ràsky E., Freidl W. (2011). The impact of socio-economic status on pain and the perception of disability due to pain. Eur. J. Pain.

